# A Retrospective Investigation: Outcome Comparisons Between Septic Patients With and Without Secondary Diagnosis of Hypothyroidism From a Rural Midwest Hospital

**DOI:** 10.7759/cureus.22467

**Published:** 2022-02-21

**Authors:** Anna Eshghi, Aaron S Fanaee, Shelly N B. Sloan, Greg Stahl, Kerry Johnson, Scott Goade, Robert Arnce

**Affiliations:** 1 College of Medicine, Kansas City University, Joplin, USA; 2 Department of Data Improvement, Freeman Health System, Joplin, USA; 3 Department of Mathematics, Missouri Southern State University, Joplin, USA; 4 Department of Pharmacy, Freeman Health System, Joplin, USA

**Keywords:** midwest, sex, mortality, hypothyroidism, sepsis

## Abstract

Background

Sepsis morbidity and mortality rates have remained high despite recent developments in clinical guidelines aimed to curtail this disease process. Understanding how sepsis interacts with comorbidities and pre-existing disease states is necessary for improving sepsis treatment. Accounting for specific pre-existing conditions in the treatment of sepsis patients may not only improve patient outcomes but also reduce healthcare costs by preventing possible complications. We sought to evaluate whether the presence of hypothyroidism affects outcomes in septic patients.

Methods

In this retrospective observational study, we analyzed the patient dataset from a not-for-profit rural hospital from January 2019 through June 2020. We chose the initial patient sample based on International Classification of Disease (ICD10) codes for sepsis. We then used the ICD10 code for hypothyroidism within that sample to identify the septic patients with hypothyroidism. We did two-sample proportion summary hypothesis tests to identify differences in mortality and 30-day readmission rates.

Results

In our dataset, we had 1,122 patients with sepsis, of whom 225 had hypothyroidism. There was no difference in sepsis outcomes between patients who had hypothyroidism compared to patients who did not have hypothyroidism. Additionally, we did not find sufficient evidence to conclude that the patient’s sex affects sepsis outcomes in hypothyroid patients.

Conclusion

Within this Midwest population, the sepsis outcomes were not impacted by having hypothyroidism as a secondary diagnosis. Additionally, there was no sufficient evidence to suggest an impact on sepsis outcomes based on sex, either male or female, when considering concomitant hypothyroidism.

## Introduction

Hypothyroidism is defined as having a thyroid gland disorder that leads to a decreased synthesis of thyroid hormones [1]. The National Health and Nutrition Examination Survey (NHANES III) reported that 4.6% of the US population has hypothyroidism [2]. The prevalence of hypothyroidism depends on many factors, with race and sex being the most significant ones. White and Mexican Americans are the most affected [2]. When evaluating sex, it has been shown that females are more commonly affected than males, specifically in the age groups 50-59 and 60-69 [2]. The clinical symptoms of hypothyroidism are non-specific and not helpful for diagnosis. Patients have complained of fatigue, weight gain, and hair loss, all of which can be symptoms of other diseases. The American Thyroid Association recommends that both sexes be screened every five years, starting at age 35 [3]. Yet, 13 million Americans have undiagnosed hypothyroidism, making it an important field of study [4]. The severity of symptoms correlates with dysfunction. However, elderly patients tend to experience less severe symptoms [1,5]. The overall mortality risk caused by hypothyroid disease remains uncertain.

Similar to hypothyroidism, sepsis affects a wide variety of people. Those at a higher risk of developing infections may also be at a higher risk of developing sepsis. This includes the very young, the very old, those with weakened or impaired immune systems, and those with chronic illnesses such as HIV or cancer. Sepsis is a common medical emergency, with 1.7 million Americans developing it every year [6]. An observational study found that sepsis causes as many deaths annually as acute myocardial infarction. The same study estimated that the incidence of sepsis would increase annually by 1.5% [7]. The population of people developing sepsis is very diverse. However, epidemiological studies have looked at many potential risk factors for sepsis. Martin et al. investigated race and found a higher incidence in African Americans, and Barnato et al. described a low incidence in Hispanics [8,9]. When looking at age, sepsis incidence increases slowly in people aged 60-64 and starts to increase rapidly in people over 85 years old [7]. Sex has been found to discriminate against sepsis as well. When age is adjusted, females have a lower risk of developing sepsis [7,10]. It was found that differences in the immune system attribute to a better prognosis for females in surgical septic patients [11]. There are conflicting results as to whether sex has an impact on mortality. However, females had a higher rate of mortality when looking at ICU admitted patients with severe sepsis [11]. 

Previous studies have shown that patients with sepsis will develop hypothyroidism secondary to the infectious process and the body's compensatory response [12,13]. However, there is a gap in knowledge regarding how patients diagnosed with hypothyroidism respond to sepsis in terms of mortality and readmission outcomes. The purpose of this study is to compare the mortality outcomes of septic patients with and without hypothyroidism to see if there is a difference. Given the sex disparities in sepsis and hypothyroidism, we also wanted to analyze patients based on sex and whether having hypothyroidism affects their sepsis outcome. This paper serves to help fill the gap in our understanding of the sepsis disease process in hypothyroid patients and provide clinicians with empirical data that can be used to help tailor the treatment of patients with hypothyroidism in the future.

## Materials and methods

This retrospective observational study was designed to investigate the impact of hypothyroidism on sepsis outcomes and analyze outcomes using sex as a variable. The study was approved by the Institutional Review Board of Freeman Health System under the protocol title: The Surviving Sepsis Campaign and its Effect on Patient Populations with Sepsis and Pre-existing Comorbidities. Due to its retrospective nature, informed consent was not required. We collected a patient dataset from the electronic medical records system of Freeman Health System, a not-for-profit rural hospital located in Joplin, MO, from January 2019 to June 2020. We chose our initial patient sample using International Classification of Disease (ICD10) diagnosis codes for sepsis listed in Table 1, which yielded 1,122 patients. Using hypothyroidism ICD10 code E03.9, we identified all patients in our initial sample who had hypothyroidism, unspecified, as a secondary diagnosis. This resulted in 225 patients leaving, and 897 patients who did not. In the hypothyroidism group, there were 143 females and 82 males (Table 2). We used two-sample proportion summary hypothesis tests to compare mortality and 30-day readmission rates between the hypothyroidism patient group and the non-hypothyroidism patient group, looking at sex as a variable. For patients in the hypothyroidism group, we used sex as a variable to compare mortality rates only. Data was insufficient for age category comparisons and further addressed in the limitations. Although our purpose was to analyze whether sex makes a difference in sepsis outcome, we did also compare males to females in the hypothyroidism group and non-hypothyroidism group, and no significance was detected.

**Table 1:** ICD10 inclusion criteria for all patients. ICD: International Classification of Disease.

ICD-10 Code	Diagnosis
A400	Sepsis due to Streptococcus, group A
A401	Sepsis due to Streptococcus, group B
A403	Sepsis due to Streptococcus pneumonia
A408	Other streptococcal sepsis
A409	Streptococcal sepsis, unspecified
A4101	Sepsis due to methicillin susceptible *S. aureus*
A4102	Sepsis due to methicillin resistant *S. aureus*
A411	Sepsis due to other specified *Staphylococcus*
A412	Sepsis due to unspecified *Staphylococcus*
A413	Sepsis due to Haemophilus influenza
A414	Sepsis due to anaerobes
A4150	Gram-negative sepsis, unspecified
A4151	Sepsis due to *Escherichia coli *(*E. coli*)
A4152	Sepsis due to *Pseudomonas*
A4153	Sepsis due to *Serratia*
A4159	Other gram-negative sepsis
A4181	Sepsis due to *Enterococcus*
A4189	Other specified sepsis
A419	Sepsis, unspecified organism

**Table 2:** Patient sample groups.

Patient group	Number of patients
Sepsis	
Male	574
Female	548
Total	1,122
Sepsis and hypothyroidism	
Male	82
Female	143
Total	225
Age and hypothyroidism	
18-39 Male	0
18-39 Female	1
40-64 Male	26
40-64 Female	38
65+ Male	56
65+ Female	104

## Results

From our total sepsis dataset, a total of 225 septic patients (20%) had hypothyroidism as a secondary diagnosis. Using a two-sample proportion summary hypothesis test, we found no significant difference in mortality or 30-day readmission rate outcomes when comparing the total hypothyroid septic group to the total non-hypothyroid septic group (Table 3, p-value <0.05 used). Results were also insignificant when comparing sex within the two groups to one another (Table 4). No evidence was observed that indicates sex influences sepsis mortality outcomes in patients with a secondary diagnosis of hypothyroidism (Table 4).

**Table 3:** Comparison of mortality and 30-day readmission of hypothyroid patients to non-hypothyroid. Two sample proportion summary hypothesis test. p1 : proportion of successes for hypothyroid population, p2: proportion of successes for non-hypothyroid population, p1−p2: difference in proportions, H0: p1−p2 = 0, HA: p1−p2 ≠ 0. HA: alternative hypothesis; Std. err: standard of error; Z-stat: standard score.

Populations	Difference	Count 1	Total 1	Count 2	Total 2	Sample difference	Std. err.	Z-stat	P-value
Mortality									
Male	p1−p2	16	82	107	492	−0.022,357,724	0.048,943,543	−0.456,806,4	0.647,8
Female	p1−p2	39	143	84	405	0.065,319,865	0.040,584,595	1.609,474,4	0.107,5
Total	p1−p2	55	225	191	897	0.031,512,449	0.030,848,617	1.021,519	0.307
30-Day readmission									
Male	p1−p2	13	83	55	492	0.046,747,967	0.038,546,441	1.212,77	0.225,2
Female	p1−p2	21	143	50	405	0.023,396,357	0.032,666,496	0.716,218,74	0.473,9
Total	p1−p2	34	225	105	897	0.034,054,255	0.024,564,066	1.386,344,4	0.165,6

**Table 4:** Comparison of mortality in female hypothyroid patients to male hypothyroid patients. Two sample proportion summary hypothesis test. p1: proportion of successes for female population, p2: proportion of successes for male population, p1−p2: difference in proportions, H0: p1−p2 = 0, HA: p1−p2 ≠ 0. HA: alternative hypothesis; Std. err: standard of error; Z-stat: standard score.

Population	Difference	Count 1	Total 1	Count 2	Total 2	Sample difference	Std. err.	Z-stat	P-value
Total	p1−p2	39	143	16	82	0.077,605,32	0.059,530,51	1.303,622,7	0.192,4

## Discussion

In this study, we first wanted to analyze whether having hypothyroidism as a secondary diagnosis impacts outcomes in patients with sepsis. When looking at mortality and 30-day readmission rate, we found these outcomes were not impacted by a secondary hypothyroidism diagnosis. In our hypothyroid population, we had 143 females (64%) and 82 males (36%) (Table 2). This difference, as well as past studies that have implicated sex as having an effect on both hypothyroidism and sepsis individually, led us to investigate the effect of sex in septic patients with hypothyroid disease. Our results suggest that sex, at least for our sample group, does not affect sepsis outcomes in patients with hypothyroidism. Despite the fact that for both diseases, sepsis and hypothyroidism, females have been shown to be more commonly affected. However, having both did not impact the outcomes for the specific sample group investigated. Our results do not mean there are no differences. However, none were found in this sample using the statistical tests performed. There may or may not be a difference for the general population as a whole.

## Conclusions

The current study revealed that septic patients with hypothyroidism do not have statistically significant differences in mortality or 30-day readmission outcomes when compared to septic patients who do not have hypothyroidism. In addition, we found no statistically significant differences in mortality outcomes based on sex in septic patients with hypothyroidism. Further studies should include age as a variable in the analysis as well as focus on a population that is relatively homogenous in terms of medical history. Additionally, a multi-center study would be able to achieve generalizable results compared to our single-center retrospective study. Due to the retrospective nature of this study, we did not have access to the patient’s hypothyroidism diagnosis and management. Further studies should categorize hypothyroidism into different types, such as primary or subclinical hypothyroidism, to see if sepsis is influenced by having a specific type of hypothyroidism. Besides diagnostic classifications, it would be beneficial to know the patient’s treatment plan to evaluate how well the hypothyroidism is controlled and compare sepsis outcomes based on mild, moderate, or severe secondary diagnosis.

Limitations

Our sample groups come from a not-for-profit rural hospital located in Southwest Missouri that serves the four-state area of Arkansas, Kansas, Oklahoma, and Missouri. Because of this, our results may not apply to other populations outside of the Midwest, including urban or suburban populations. Our population was not randomly selected and had a low sample size overall. There was great variability between the sizes of our two groups, with our hypothyroid group being almost four times smaller than our non-hypothyroid group. We selected our initial sepsis sample group for severe illness and ICD10 codes specific to sepsis, which were charted at the patient’s visit. The ICD10 for hypothyroidism was charted as a new diagnosis at the same visit. Hence, our sample population was not randomly selected and may not be representative of their respective populations as a whole. It is also important to note that our patient population is heterogeneous, as they likely have multiple other diseases, including various secondary comorbidities affecting their health and overall septic outcome. These confounding variables were not considered in our analysis. Hence, it is valid to assume that the other comorbidities could have played a significant role in this group’s patient sepsis outcomes, but their analysis is outside of this study. Our data set did not allow us to define a personal history of hypothyroidism as patient charts and secondary comorbidities listed were specific to the original screen for sepsis ICD10 codes (Table 1). It is assumed all patients screened using ICD10 code E03.9 would have received that diagnosis upon the patients visit for sepsis. We did not have access to the patient’s medical history. Due to the retrospective nature of this study, we did not have the ability to further categorize hypothyroidism based on type, treatment plan, or severity. We were unable to obtain any medication profiles from our patients. Hence, we were unable to determine how well their hypothyroidism was being managed and what the severity is. In addition, electronic medical records did not include any lab work or screening results for subcategories such as Euthyroid sick syndrome, subclinical hypothyroidism, or medications. This means we did not have access to the type of hypothyroidism, what medications they were taking, or how long they had been treated for it. Although myxedema is included in the ICD10 codes for hypothyroidism, we do not know which patient had it. As a result, we were unable to further classify our patients into specific categories. Additionally, our patients were categorized into the age groups of 18-39, 40-64, and 65 or greater (Table 2). However, we did not have an equal number of patients in each age group. Thus, for our analysis, we did not use age as a variable for outcomes. Therefore, it is unclear if age as a variable would have changed our results.
